# Grade Prediction of Bleeding Volume in Cesarean Section of Patients With Pernicious Placenta Previa Based on Deep Learning

**DOI:** 10.3389/fbioe.2020.00343

**Published:** 2020-04-30

**Authors:** Jun Liu, Tao Wu, Yun Peng, Rongguang Luo

**Affiliations:** ^1^Department of Information Engineering, Nanchang Hangkong University, Nanchang, China; ^2^NuVasive, San Diego, CA, United States; ^3^Department of Medical Imaging and Interventional Radiology, The First Affiliated Hospital of Nanchang University, Nanchang, China

**Keywords:** image recognition, deep learning, image segmentation, MRI uterus image, assistant diagnosis

## Abstract

In order to predict the amount of bleeding in the cesarean section of the patients with Pernicious Placenta Previa (PPP), this study proposed an automatic blood loss prediction method based on Magnetic Resonance Imaging (MRI) uterus image. Firstly, the DeepLab-V3 + network was used to segment the original MRI abdominal image to obtain the uterine region image. Then, the uterine region image and the corresponding blood loss data were trained by Visual Geometry Group Network-16 (VGGNet-16) network. The classification model of blood loss level was obtained. Using a dataset of 82 positive samples and 128 negative samples, the proposed method achieved accuracy, sensitivity and specificity of 75.61, 73.75, and 77.46% respectively. The experimental results showed that this method can not only automatically identify the uterine region of pregnant women, but also objectively determine the level of intraoperative bleeding. Therefore, this method has the potential to reduce the workload of the attending physician and improve the accuracy of experts’ judgment on the level of bleeding during cesarean section, so as to select the corresponding hemostasis measures.

## Introduction

Pernicious placenta previa is a specific type of placenta previa that occurs when the placenta attaches to previous cesarean scars. PPP-induced massive hemorrhage during cesarean section has common clinical case (Yifan et al., 2017; Xiaoqin et al., 2019; Yu et al., 2019). The amount of bleeding during PPP cesarean section can typically range between 2,000 and 7,800 ml, and can even reach up to 20,000 ml. The amount of red blood cell transfusion can reach up to 50 units (Shuhong et al., 2017; Sharafi and Ghasemi, 2019; Ryu et al., 2019). The resulting rate of hysterectomy can be as high as 20.59–100%, and the maternal mortality rate as high as 7–10% (Jun et al., 2018; Qiu et al., 2018). Therefore, it is of great clinical importance to adopt a fast and effective hemostasis method to reduce the intraoperative risk.

**FIGURE 1:**
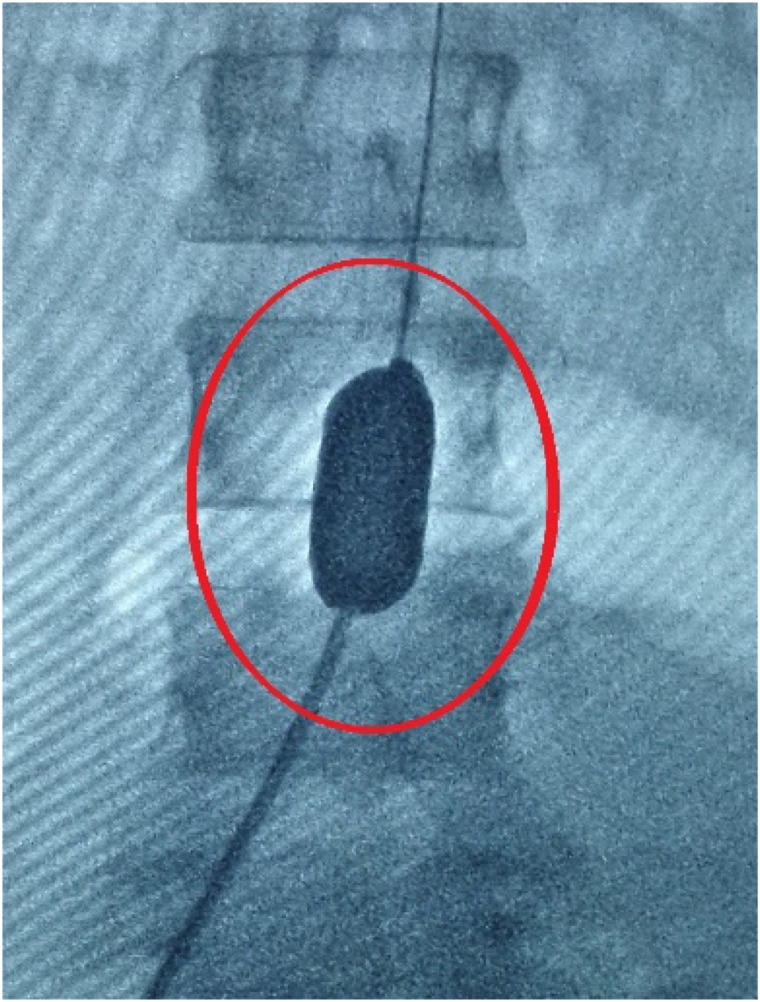
Filled intra-arterial balloon.

Endovascular Balloon Occlusion (EBO) is a commonly performed procedure to reduce the amount of bleeding during cesarean section in pregnant women with PPP (Na et al., 2018; Yongchun et al., 2018). EBO is used to prevent fatal postpartum hemorrhage by pre-positioning the balloon catheter in the infrarenal abdominal aorta (Figure 1) in the bilateral iliac crest or in the common iliac artery before cesarean section (Meng-jun et al., 2018; Na et al., 2018). This method is mainly suitable for the possibility of intraoperative, postoperative major bleeding complications or abnormal attachment of various types of placenta (placenta previa, placental adhesion, placenta implantation, penetrating placenta; Meng-jun et al., 2018). However, the EBO procedure also bears certain risks (Zheng et al., 2017; Zhu et al., 2017; Karlsen et al., 2018). First, the addition of hemostatic balloon can cause thrombosis in arteries, leading to serious complications to patients. The incidence can be as high as 10% (Shkumat et al., 2018). Second, intra-arterial balloon is often installed with guidance of Digital Subtraction Angiography (DSA), which brings a certain dose of X-ray radiation to the fetus and highly undesirable by patients (Chaoqun et al., 2018; Duracka et al., 2018; Miller et al., 2018). Therefore, it is important to accurately predict the possible amount of bleeding of the pregnant women with PPP before cesarean section, and judge the necessity to preset the intra-arterial balloon catheter to avoid the balloon catheter-associated complications.

However, the decision on whether to pre-install the catheter before operation largely relies on surgeons’ experience in interpreting medical images and clinical reports. As a result, there may be potentially high misdiagnosis and missed diagnosis (Li and Deng, 2017; Ying et al., 2019). In the existing literature, attempts have only been made to predict whether the placenta is invasive (Huaiqiang et al., 2019). For example, Zhu and Xie (2019) used the ultra-scoring system to evaluate the degree of danger during cesarean section. They found that this system could be of great value to evaluate PPP combined with PAS and poor pregnancy outcomes, enhancing relations between sonographic fndings and PAS complications. Mengjun et al. (2018) made preoperative judgment on the bleeding of patients with dangerous placenta previa after cesarean section. In another study by Mehrabadi et al. (2015), the authors studied the influence of placenta implantation on severe postpartum hemorrhage and found that placenta implantation was the main cause of postpartum hemorrhage. However, we are not aware of any existing studies that investigated the utility of deep learning in predicting the intraoperative risk of amount of bleeding. In a most recent work by Huaiqiang et al. (2019), the authors predicted whether it is an invasive placenta using a feature extraction based machine learning algorithm. However, their study did not support a direct prediction of the level of intraoperative blood loss, which can be otherwise very useful for doctors to plan preoperatively whether an EBO procedure may be necessary.

In this study, we aimed to propose a method to predict the degree of intraoperative bleeding based on MRI uterus image and deep learning technology for the cesarean section of the pregnant woman with PPP. To the best of our knowledge, this is the first study to use deep learning method and uterine MRI images to predict the level of bleeding during cesarean section in patients with PPP.

## Materials and Methods

### Data Collection

**FIGURE 2:**
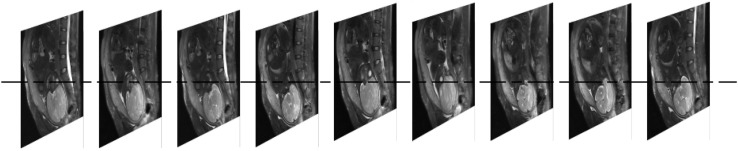
Each patient contains 9 layers of MRI sequence images.

The amount of bleeding is often classified as high (bleeding volume > 500 ml; preoperative hemostasis needed) or low (bleeding volume < 500 ml; only routine hemostasis) (Yan and Yan, 2017). In this study, the data was collected from the First Affiliated Hospital of Nanchang University, including 210 cases of low-grade bleeding determined by doctors subjectively, without hemostasis plan taken before operation (Supplementary Material). All MRI images were taken within one week before delivery on a 1.5T clinical scanner (Siemens Medical System). Eighty two patients had a bleeding volume =500 ml (positive cases). One hundred and twenty eight patients had a bleeding volume <500 ml (negative cases) in actual surgery. Each case contained 9 MRI sequence images with a layer spacing of 7 mm (Figure 2). The resolution of each MRI image is 384 by 512px. In order to improve the calculation efficiency, all MRI sequences were transformed to Joint Photographic Experts Group (JPEG) format with a gray level of 256. The operation report of each case included the information related to the experiment, such as the amount of bleeding during the operation and whether to use hemostatic balloon or not. Table 1 shows the age distribution of patients. In order to expand the amount of image data, each pregnant woman’s 9 MRI images are used separately as 9 independent cases (de Vos et al., 2019; Mathieu et al., 2019).

**TABLE 1:** Age distribution of patients (*p* = 0.97, Chi-Square test).

Age	Total	Negative	Positive
16–25	34	22	12
26–35	125	77	48
36–45	51	29	22

### Methods

#### Automatic Segmentation of the Cervical Area

**FIGURE 3:**
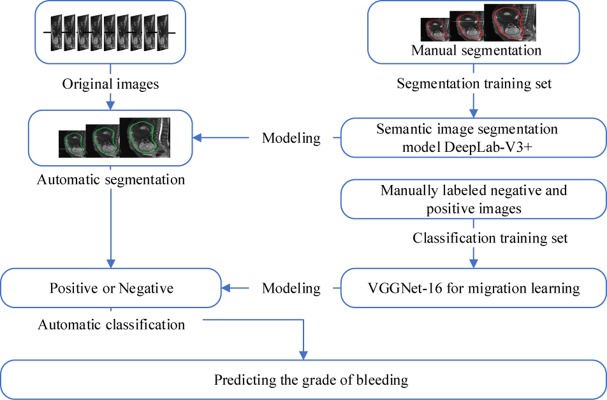
Flow chart of experimental method.

**FIGURE 4:**
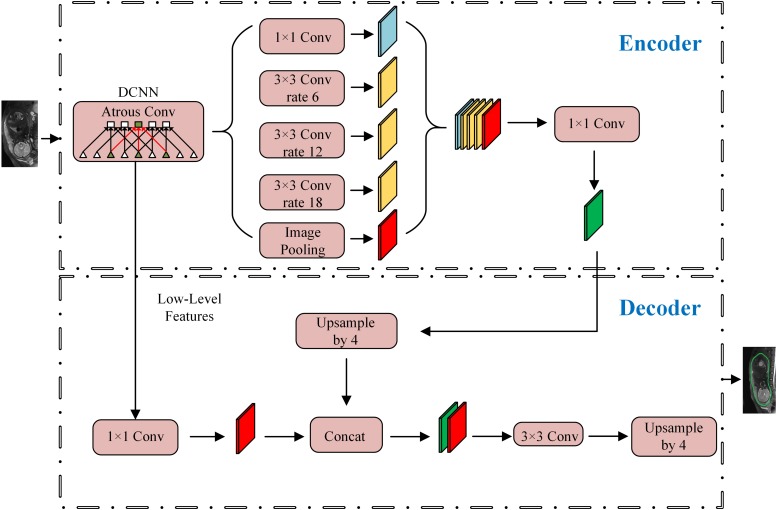
Structure chart of DeepLab-V3 +.

The overall workflow is shown in Figure 3. The first step was to segment the uterine region from the original MRI image using DeepLab-V3 + network (Figure 4). The purpose of this step was to avoid the influence of background region on the subsequent feature extraction and classification (Ming et al., 2019; Song et al., 2019). DeepLab-V3 + is a semantic segmentation method. Compared with image classification or target detection, semantic segmentation allows for a more detailed understanding of images, which is important in applications such as autopilot, robots and image search engines. Although unsupervised methods such as clustering can be used for segmentation, the results are not necessarily semantic.

A total of 210 images were used in the segmentation experiment, which were randomly selected from 9 sequence images of each case. A 5-fold cross validation was used, with 168 segmented images were used for training, and the remaining 42 were used for testing, and repeated for eight times. The gold standard of segmentation of 210 sample images was obtained by doctors’ manual segmentation. Five experienced obstetricians (experts) participated in the study. Each image was segmented by one expert and confirmed by the other four experts.

The construction of DeepLab-V3 + network was completed on the TensorFlow platform. During the training, two images were input at a time, and the number of training rounds was set to 400. All 210 images were input in each round. The learning rate of the network was set to 10^–6^, and the other parameters remained the default value of TensorFlow platform (Mukhoti and Gal, 2018; Yujie et al., 2018). The segmented region was operated by a circular expansion operator with a radius of 5px to avoid the loss of texture information of key boundaries, as there may be texture information and other morphological features in the boundary. Equation (1) is the morphological operation:

(1) $$A⊕B=\left\{a|{\left(B\right)}_{a}⋂A\neq \emptyset \right\}$$

where *A* is the segmented region and *B* is the expansion structure operator with a radius of 5px.

When the morphological operation was completed, the image of the segmented uterine region was extracted by the method of the smallest rectangular box. Then the image was scaled to the retained scale, making the minimum edge 224px, and saved as the sample image for the subsequent construction of the bleeding level classification model.

After the semantic segmentation was completed, the performance of the segmentation was analyzed using the accuracy, sensitivity and specificity.

(2) $$Accuracy=\frac{TN+TP}{TN+TP+FN+FP}$$

(3) $$Sensitivity=\frac{TP}{TP+FP}$$

(4) $$Specificity=\frac{FN}{TN+FN}$$

where True Positive (*TP*) is the number of pixels that belonged to the uterus region and also accurately labeled so, True Negative (*TN*) is the number of pixels that belonged to a non-uterus region and also accurately labeled so, False Positive (*FP*) is the number of pixels that belonged to a non-uterus region, but falsely labeled as uterus, and False Negative (*FN*) is the number of pixels that belonged to the uterus region but falsely labeled as a non-uterus region.

In the experiment, Dice Similarity Coefficient (*DSC*) was also used to evaluate the segmentation performance:

(5) $$DSC=\frac{2\left|R_{a}\cap R_{b}\right|}{\left|R_{a}\right|+\left|R_{b}\right|}$$

where *R*_*a*_ and *R*_*b*_ represent the automatic segmentation area and manual segmentation area respectively and symbol “| | ” means the calculation of the number of pixels.

#### Deep Learning Based Classification of Bleeding Volume

**FIGURE 5:**
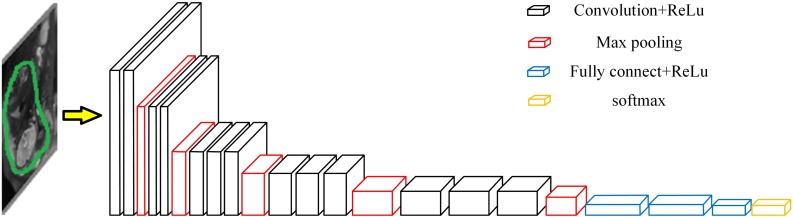
Structure chart of VGGNet-16.

After removing the background from the uterus region image, according to the uterus region image and the corresponding bleeding volume level data (negative cases or positive cases), a bleeding volume level classification model was developed using VGGNet-16 network with Transfer Learning (Abidin et al., 2018; Zhe et al., 2019). The framework is shown in Figure 5 (Lin and Yuan, 2019).

Because the scale of positive and negative cases in this project is not equal, this study took a small number of positive cases as the benchmark. For positive cases, each experiment randomly selected 70 cases of 82 cases as training samples, and the remaining 12 cases as test samples. For the negative cases, 70 cases of training samples were randomly selected from 128 cases in each experiment, and 12 of the remaining cases were randomly selected as test samples. Each case contained 9 images as an independent sample to participate in the training and testing, so there were 630 negative and 630 positive images as training samples and 108 negative and 108 positive images as test samples in each experiment.

The experiment was conducted on the TensorFlow platform. The number of training steps for the VGGNet-16 network was set to 2 × 10^4^, the learning rate was set to 10^–2^, and the remaining parameters were retained as default values for the TensorFlow platform (Rui et al., 2019). The parameters of two models were chosen by using trial-and-error method according to Abidin et al. (2018) and Song et al. (2019). The training and testing process were repeated 100 times in the manner described above to observe the generalization performance of the model. In order to further observe the influence of segmentation of uterine region on the accuracy of classification model, the experiment repeated the above experiments using the original image, the image of uterine region after segmentation by doctors and the image of uterine region segmented by DeepLab-V3 + network model as the input image.

In the same way, the experiment uses Equations (2)–(4) to analyze the performance of classification model. In this case, the True Positive (*TP*) represents the number of high blood loss cases that were correctly classified so, the True Negative (*TN*) represents the number of low blood loss cases that were correctly classified so, the False Positive (*FP*) represents the number of low blood loss cases falsely classified as high blood loss cases, and the False Negative (*FN*) represents the number of high blood loss cases falsely classified as low blood loss cases.

#### Comparison Experiments

In order to verify the effectiveness of this scheme, experiments were also performed to compare to the results of a previous study by Huaiqiang et al. (2019). The experiment first selected 108 images with positive bleeding volume and 108 negative blood volumes for Laplacian of Gaussian (LoG)-filtered in the data set, and then extracted features for the filtered images. As shown in Table 2, the experiment extracted the top 10 features with the most predictive power. The remaining features contribution ratio was <3% The Gradient Boosting Classifier was used to classify the bleeding volume level, and the experiment was repeated 100 times.

**TABLE 2:** The top 10 features with the most predictive power.

No.	Feature
1	GLCM Correlation [LoG filtered (σ = 1 mm) Turbo Spin Echo (TSE)] (Li et al., 2019)
2	GLSZM Zone Entropy [LoG filtered (σ = 1 mm) TSE] (Hoang et al., 2018; Zhen et al., 2018)
3	GLSZM Short Run Low Gray Level Emphasis [LoG filtered (σ = 5 mm) TSE] (Hoang et al., 2018)
4	GLSZM Zone Percentage [original balanced turbo field echo (bTFE)] (Hoang et al., 2018; Zhen et al., 2018)
5	GLCM Inverse Variance [LoG filtered (σ = 5 mm) TSE] (Zhen et al., 2018)
6	GLRLM Run Entropy [LoG filtered (σ = 3 mm) TSE] (Zhen et al., 2018)
7	GLDM Small Dependence High Gray Level Emphasis [LoG filtered (σ = 5 mm) TSE] (Zhen et al., 2018)
8	GLCM Difference Entropy [LoG filtered (σ = 5 mm) TSE] (Hoang et al., 2018)
9	GLSZM Zone Entropy [LoG filtered (σ = 3 mm) TSE] (Hoang et al., 2018)
10	GLSZM Gray Level Non-Uniformity (original TSE) (ZhiCheng et al., 2019)

## Results

### Automatic Segmentation of Cervical Regions

**FIGURE 6:**
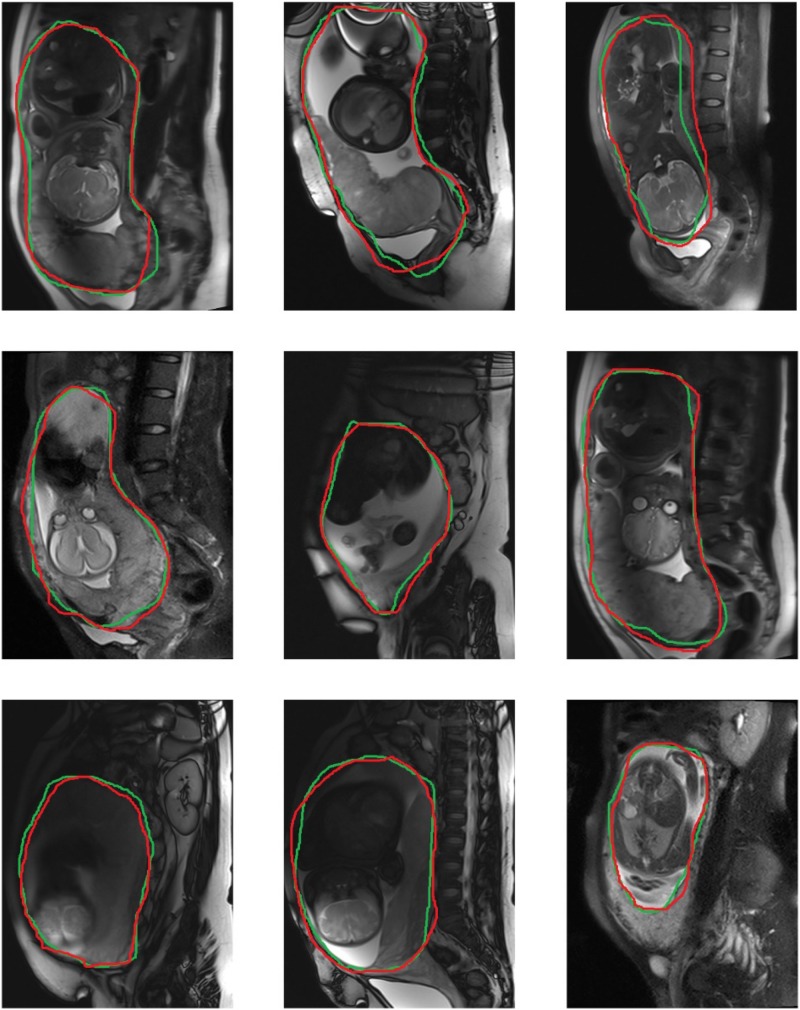
Representative diagrams of segmentation boundary results, with green curves representing automatic segmentation, and red curves manual segmentation.

**FIGURE 7:**
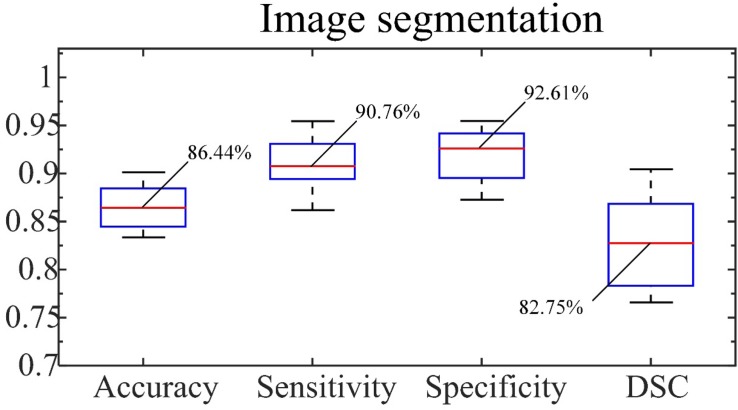
Accuracy, sensitivity and specificity of segmentation and DSC. The number indicated is the median.

From representative images of the manual (ground truth) and automatic segmentation (Figure 6), the automatic segmentation showed satisfying results that are close to the ground truth, achieving an accuracy of 86.44%, sensitivity of 90.76%, and specificity of 92.61%. The median of DSC is 82.75% (Figure 7).

### Classification of Bleeding Volumes

**FIGURE 8:**
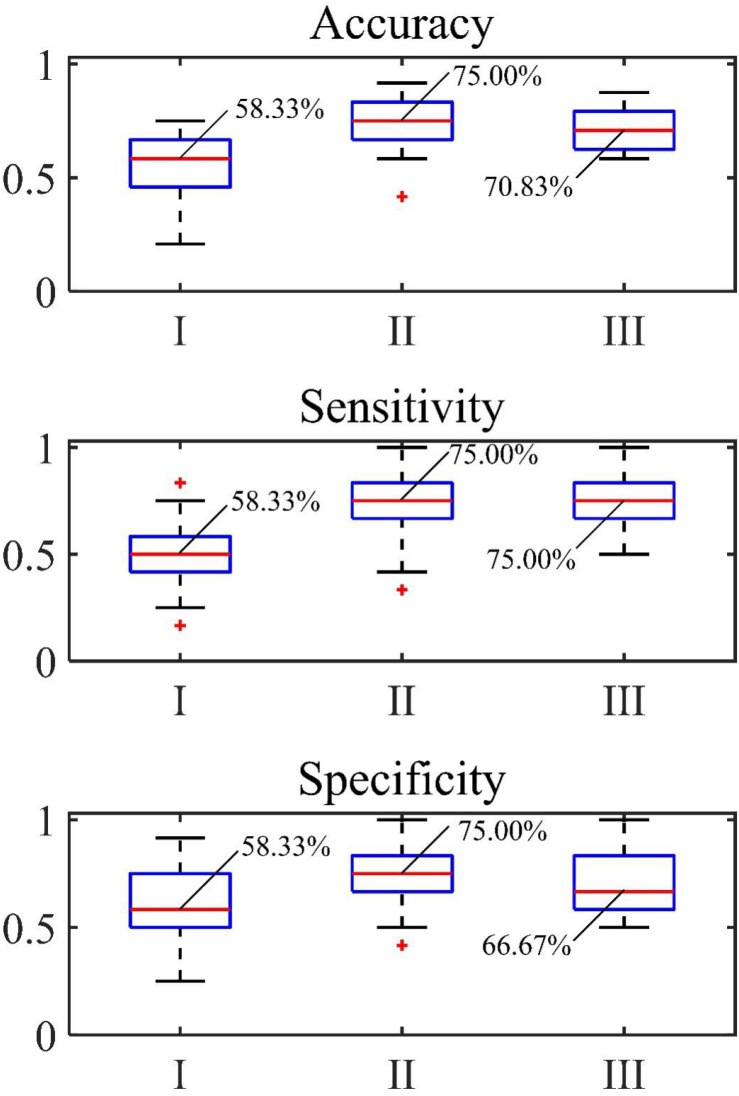
Accuracy, sensitivity and specificity of classification of (I) original image, (II) automatic segmentation and (III) manual segmentation.

**FIGURE 9:**
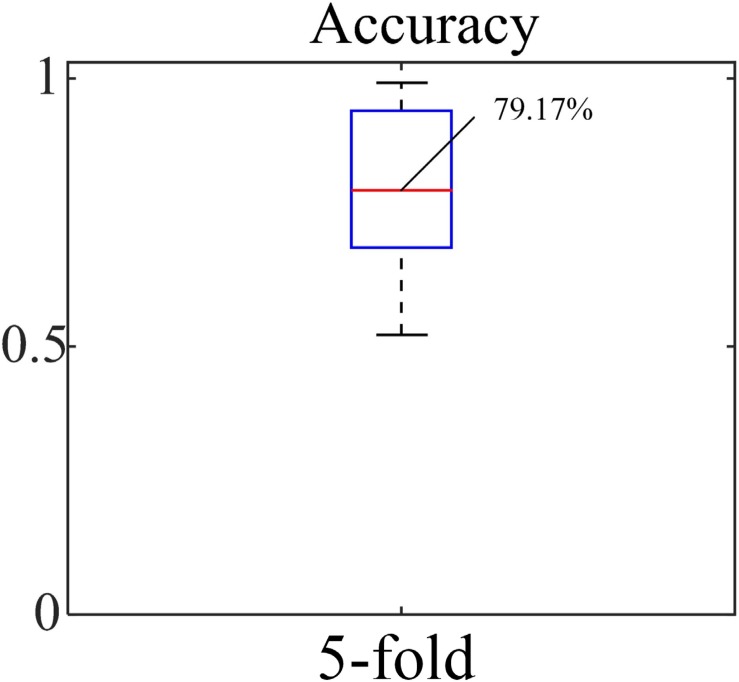
Average accuracy by 5-fold cross validation.

The accuracy, sensitivity and specificity for the blood loss volume classification are shown in Table 3 and Figure 8 for all three segmentation methods (none, manual or automatic). If no segmentation was used, the classification showed an accuracy of 56.71%, sensitivity of 58.94%, and specificity of 54.47%. Using manual segmentation, the classification performance was markedly improved, showing an accuracy of 72.59%, sensitivity of 74.15%, and specificity of 71.02%. When the automatic segmentation (DeepLab-V3 + network) was used, the classification showed an accuracy of 75.61%, sensitivity of 73.75% and specificity of 77.46%. Interestingly, the classification accuracy achieved by the automatic segmentation, compared to using manual segmentations, was further improved by 3.02%. A 5-fold cross validation was performed eight times. It showed that the average accuracy of the model is 78.93% and the median is 79.17% (Figure 9).

**TABLE 3:** Quantitative statistics of classification performance after three segmentation processes.

Performance index	Accuracy		Sensitivity		Specificity	
	Average	Median	Average	Median	Average	Median
No segmentation	56.71%	58.33%	58.94%	58.33%	54.47%	58.33%
Manual segmentation	72.59%	70.83%	74.15%	75.00%	71.02%	66.67%
Automatic segmentation	75.61%	75.00%	73.75%	75.00%	77.46%	75.00%

### Comparison Experiment

**FIGURE 10:**
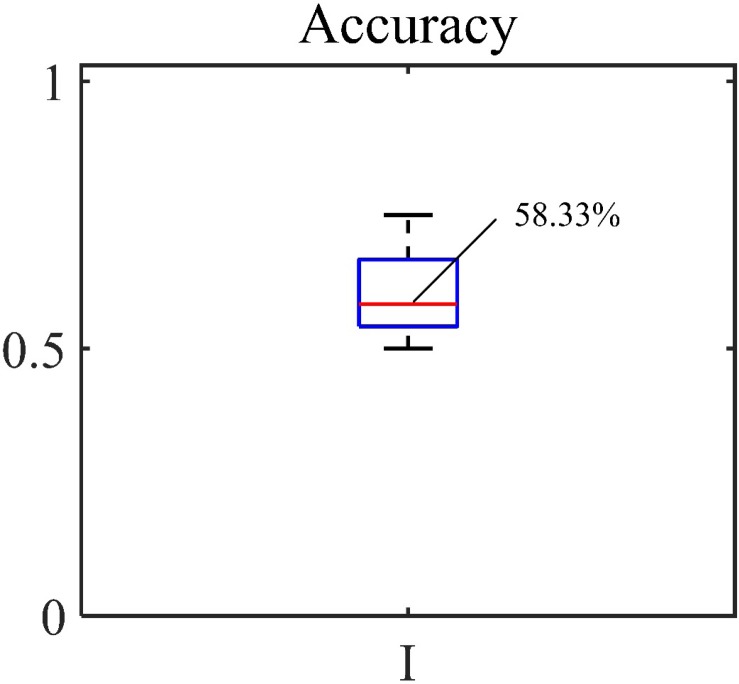
Accuracy of the comparison experiment.

Gradient Boosting Classifier was used to classify the bleeding volume level, and the accuracy of the experiment was calculated. The statistical results are shown in Figure 10. The average accuracy rate is 61.01%. The accuracy of Sun’s top 10 features is slightly lower than that of the model constructed in this paper.

## Discussion

In this study, a deep learning method based on MRI uterus images was developed to automatically segment cervical areas and then predict intraoperative blood loss level. Given the lack of an automated method for this purpose, this method can assist in the preoperative plan for hemostasis. The results showed that automatic segmentation can accurately reproduce the segmentation results manually performed by doctors. Furthermore, the classification results based on automatic segmentation showed the best performance. To the best of our knowledge, this is the first study to use deep learning method and uterine MRI images to predict the level of bleeding during cesarean section in patients with PPP.

Previous clinical studies have shown that the volume of bleeding is associated with a number of morphologic features, such as the shape/orientation of the placenta/lesion (Huaiqiang et al., 2019). It is our hypothesis that these unique features can be reflected on MR images, and more importantly, can be effectively learned by the deep learning model by the convolution operation with proper training (which is a known strength of deep learning in imaging analysis). Our results appeared to have confirmed this hypothesis. In terms of classification results, the average accuracy rate of this experiment achieved 75.60%. It is worth noting that, this accuracy was greater than the prediction (an accuracy of 61%) made prior to the surgery by the attending doctors (in this study, 210 cases were judged to have low bleeding volumes by the doctors preoperatively, but only 128 of them turned out to be accurate after surgery).

Accurate segmentation of the uterus area plays a critical role to the success of classification. When no segmentation was performed, the classification performance was the worst. This finding suggested that the background area may contain information that interfered with the classification. Therefore, removing background through a proper segmentation appeared an important step to consider. Interestingly, it was found that the classification performance when using the automatic segmentation method was in fact improved compared to that of the manual segmentation (the gold standard of segmentation). This can be potentially due to the inclusion of an area on the MRI image that surround the actual boundary of the uterus, which may better capture the physiological information (e.g., contrast) at the boundary. Therefore, for the classification of bleeding volumes, a middle ground appeared to exist in terms of segmentation between non-segmentation and “perfect” segmentation (no inclusion of background area in the immediate proximity of the anatomical boundary of the uterus).

This study has several limitations. First, a relatively small sample size was used. Second, this study was based on a single institute. Lastly, only MR images were used as the sole inputs for classification. For future research directions, it is the authors’ opinion that additional data (e.g., clinical lab test results), in addition to MR images, could be integrated to into the deep learning model to improve the accuracy, or even provide quantitative estimate of the bleeding volume (in contrast to a two-class classification in our current setup). Doing so would require a much larger dataset. Therefore, future studies based on a larger sample size (preferably from multiple institutes) that include a broader spectrum of features (e.g., patient history, demographics, clinical reports and imaging data) are warranted to further improve the deep learning model as an assistive tool to reduce the intraoperative risk for patients with PPP.

## Conclusion

A deep learning-based model was successfully developed in this study to predict the level of bleeding in cesarean section of patients with dangerous placenta previa. The results suggested that this method has the potential to predict the bleeding volume in cesarean section and serve as a clinical assistive tool to reduce the intraoperative risk for patients with PPP.

## Data Availability Statement

The datasets generated for this study are available on request to the corresponding author.

## Ethics Statement

Medical Research Ethics Committee of the First Affiliated Hospital of Nanchang University confirms that ethics approval and specific consent procedures are not required for this study. Ethical approval for the study and written informed consent from the participants of the study were not required according to national legislation and institutional requirements.

## Author Contributions

JL was responsible for guiding the experiment process. TW was responsible for the implementation of the experimental process. YP translated the manuscript. RL was responsible for data collection and medical guidance.

## Conflict of Interest

YP was a fulltime employee of NuVasive but contributed to this study independently of his employment. The remaining authors declare that the research was conducted in the absence of any commercial or financial relationships that could be construed as a potential conflict of interest.

## Supplementary Material

DATA SHEETS S1 Original images.
