# Supplementary figures and images for: Grade Prediction of Bleeding Volume in Cesarean Section of Patients With Pernicious Placenta Previa Based on Deep Learning
Source: Front Bioeng Biotechnol. 2020 Apr 30;8:343. doi: 10.3389/fbioe.2020.00343 (PMC7203465; doi:10.3389/fbioe.2020.00343)

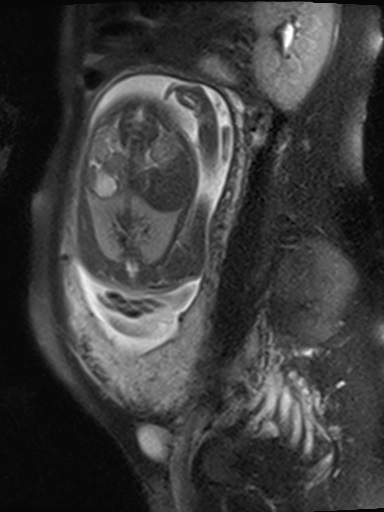

Supplement: DATA SHEETS S1 — Original images. [file Data_Sheet_1.ZIP › supplementary material/IM000076.jpg]

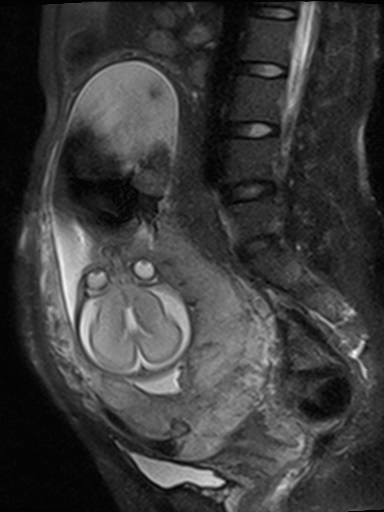

Supplement: DATA SHEETS S1 — Original images. [file Data_Sheet_1.ZIP › supplementary material/IM000087.jpg]

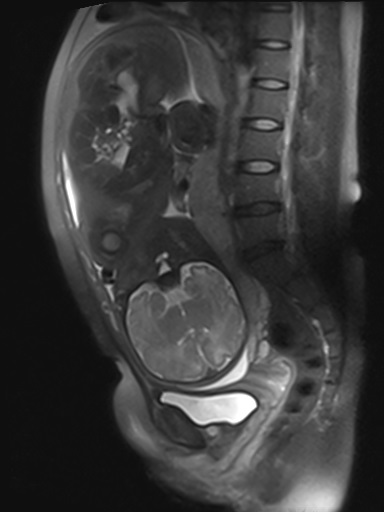

Supplement: DATA SHEETS S1 — Original images. [file Data_Sheet_1.ZIP › supplementary material/IM000107.jpg]

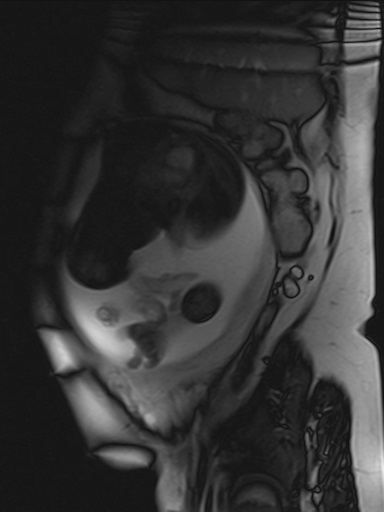

Supplement: DATA SHEETS S1 — Original images. [file Data_Sheet_1.ZIP › supplementary material/IM000141.jpg]

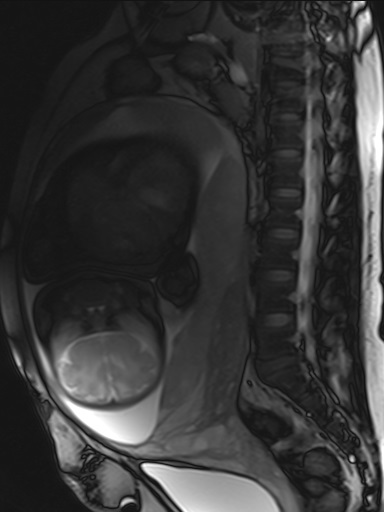

Supplement: DATA SHEETS S1 — Original images. [file Data_Sheet_1.ZIP › supplementary material/IM000149.jpg]

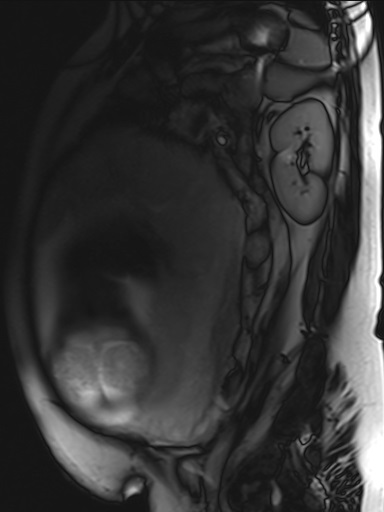

Supplement: DATA SHEETS S1 — Original images. [file Data_Sheet_1.ZIP › supplementary material/IM000154.jpg]

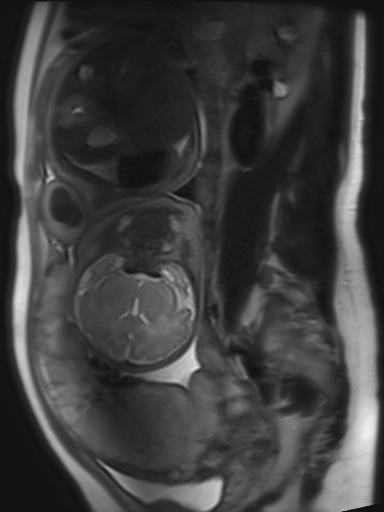

Supplement: DATA SHEETS S1 — Original images. [file Data_Sheet_1.ZIP › supplementary material/IM000176.jpg]

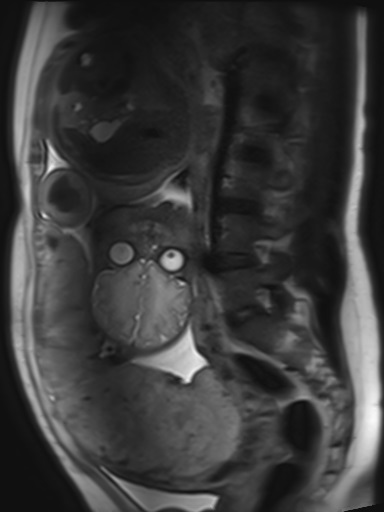

Supplement: DATA SHEETS S1 — Original images. [file Data_Sheet_1.ZIP › supplementary material/IM000179.jpg]

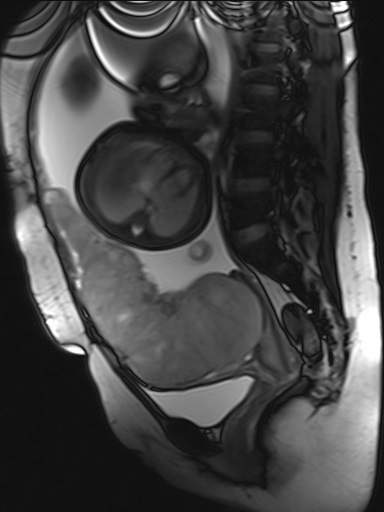

Supplement: DATA SHEETS S1 — Original images. [file Data_Sheet_1.ZIP › supplementary material/IM000188.jpg]
